# Preparation of Defined Chitosan Oligosaccharides Using Chitin Deacetylases

**DOI:** 10.3390/ijms21217835

**Published:** 2020-10-22

**Authors:** Martin Bonin, Sruthi Sreekumar, Stefan Cord-Landwehr, Bruno M. Moerschbacher

**Affiliations:** Institute for Biology and Biotechnology of Plants, University of Münster, 48143 Münster, Germany; martin.bonin@uni-muenster.de (M.B.); sruthi.sreekumar@uni-muenster.de (S.S.); stefan.cord-landwehr@uni-muenster.de (S.C.-L.)

**Keywords:** paCOS, chito-oligosaccharide, CDA, chitin deacetylase, pattern of acetylation, chitosan, chitin, chitinase, chitosanase, regio-selective

## Abstract

During the past decade, detailed studies using well-defined ‘second generation’ chitosans have amply proved that both their material properties and their biological activities are dependent on their molecular structure, in particular on their degree of polymerisation (DP) and their fraction of acetylation (*F*_A_). Recent evidence suggests that the pattern of acetylation (PA), i.e., the sequence of acetylated and non-acetylated residues along the linear polymer, is equally important, but chitosan polymers with defined, non-random PA are not yet available. One way in which the PA will influence the bioactivities of chitosan polymers is their enzymatic degradation by sequence-dependent chitosan hydrolases present in the target tissues. The PA of the polymer substrates in conjunction with the subsite preferences of the hydrolases determine the type of oligomeric products and the kinetics of their production and further degradation. Thus, the bioactivities of chitosan polymers will at least in part be carried by the chitosan oligomers produced from them, possibly through their interaction with pattern recognition receptors in target cells. In contrast to polymers, partially acetylated chitosan oligosaccharides (paCOS) can be fully characterised concerning their DP, *F*_A_, and PA, and chitin deacetylases (CDAs) with different and known regio-selectivities are currently emerging as efficient tools to produce fully defined paCOS in quantities sufficient to probe their bioactivities. In this review, we describe the current state of the art on how CDAs can be used in forward and reverse mode to produce all of the possible paCOS dimers, trimers, and tetramers, most of the pentamers and many of the hexamers. In addition, we describe the biotechnological production of the required fully acetylated and fully deacetylated oligomer substrates, as well as the purification and characterisation of the paCOS products.

## 1. Why Using Defined Chitosans Is so All-Important in Bioactivity Studies

The term ‘chitosan’ describes a large and versatile family of oligo- and polysaccharides with different structures and multiple functions. Some chitosans have antimicrobial activity, inhibiting bacterial growth as well as germination, growth and development of fungi and oomycetes. Some chitosans have antiviral activity and some inhibit growth and development of nematodes. Some chitosans stimulate plant growth and development, some induce plant disease resistance, and some improve plant abiotic stress tolerance. Some chitosans support scar-free wound healing in animals and humans, some appear to inhibit tumour cell growth, some may have anti-inflammatory or anti-oxidant potential, and a plethora of other biomedically relevant bioactivities have also been reported. However, all of these biological functionalities have been notoriously unreliable. Sometimes, the above-mentioned bioactivities can be observed, and sometimes not. We have argued that we need to know molecular structure-function relationships of chitosans to achieve reliable results [1,2]. Chitosans differ in their degree of polymerisation (DP) and in their fraction of acetylation (*F*_A_), and both parameters deeply influence the physico-chemical properties and the biological activities of the polymers.

Two landmark papers by Kauss et al. [3] and Vander et al. [4] represent crucial steps in this process of understanding structure-function relationships of partially acetylated chitosan polymers. Both papers clearly indicated the crucial role of *F*_A_ and, less marked, of DP for the biological activity of different chitosans, as exemplified in their elicitor activities towards plant cells. Kauss et al. [3] reported that the elicitor activity of chitosan polymers with sufficiently high DP decreased with increasing *F*_A_, i.e., high-DP low-*F*_A_ chitosans were most active in inducing resistance reactions in *Catharanthus roseus* cells. The authors concluded that it is the polycationic character of the chitosan which is responsible for the elicitor activity, and they suggested electrostatic interactions between the polycationic polymer and its suggested target, the polyanionic phospholipid membrane, as a mode of action. We might call this the ‘target hypothesis’ of chitosans’ mode of action.

Vander et al. [4] reported that the elicitor activity of chitosan polymers with sufficiently high DP first increased, then decreased with increasing *F*_A_; very low-*F*_A_ chitosans and very high-*F*_A_ chitosans were elicitor-inactive, only high-DP medium-*F*_A_ chitosans were active in inducing resistance reactions in wheat leaves. The authors concluded that it is the combination of the partly polycationic and partly hydrophobic character of the chitosan which is responsible for the elicitor activity, and they suggested molecular recognition by a most-likely transmembrane proteinaceous, partly polyanionic and partly hydrophobic receptor as a mode of action. We might call this the ‘receptor hypothesis’ of chitosans’ mode of action.

Based on these and similar later results, the chitosan matrix was introduced to visualise the structure-function dependencies of partially acetylated chitosans, clearly indicating that different biological activities depend on different structural aspects of chitosans [1,5,6]. This marked the transition from yesterday’s first-generation raw chitosan to today’s second-generation defined chitosans. First-generation chitosan samples are poorly defined mixtures of molecules with highly different and mostly unknown DP and *F*_A_, hence with potentially huge batch-to-batch differences. Second-generation chitosans, in contrast, are well-defined in terms of their DP and *F*_A_ and, therefore, possess defined physico-chemical properties and known biological activities. Upon dissemination of this scientific knowledge, some forward-thinking commercial chitosan producers began to introduce rigorous quality control measures so that second-generation chitosans became available in good quality and at industrial scale, allowing the development of reliable chitosan-based products.

While we now know much about molecular structure-function relationships of chitosans, we are still far from understanding their cellular modes of action. The discussion of whether chitosans act by rather non-specifically interacting with target structures (i.e., the ‘target hypothesis’) or rather by specifically interacting with cognate receptor molecules (i.e., the ‘receptor hypothesis’) is ongoing. A crucial consideration here is the fact that practically all organisms appear to secrete sequence-specific chitosan hydrolases [7,8]. Most bacteria and fungi secret chitinases and/or chitosanases, and these may protect them from the antimicrobial action of chitosan polymers [9,10,11], arguing in favour of the membrane target hypothesis which requires polymers for bioactivity. In contrast, human, animal, and plant cells rather secret chitinases, but not chitosanases, and these may be essential in generating partially acetylated chitosan oligosaccharides (paCOS) which may be recognised by specific receptors. However, to date, no chitosan-specific receptor has been described in any detail, while chitin receptors are known in both humans and plants [12,13,14,15]. Perhaps, the observed elicitor activity of medium- *F*_A_ chitosans in plants can best be explained by the action of plant chitinases degrading high-DP medium-*F*_A_ chitosan polymers into low-DP low-*F*_A_ chitosan polymers and high-*F*_A_ chitosan oligomers, the former non-specifically reacting with target membranes and the latter being specifically recognised by chitin receptors [2]. In that case, both the target and the receptor hypotheses would apply.

Thus, a picture is emerging in which sequence-specific chitosan hydrolases process partially acetylated chitosan polymers, resulting in smaller, modified chitosan polymers and yielding structurally more or less defined mixtures of paCOS. In this scenario, the pattern of acetylation (PA), i.e., the distribution of acetylated GlcNAc (A) and de-acetylated GlcN (D) units in the chitosan chains, emerges as a third and crucial determinant of bioactivities. First, the different sub-site specificities and preferences of the sequence-specific chitosan hydrolases for GlcNAc or GlcN units determine at which sites-if at all-a given chitosan polymer will be cleaved, and they will concomitantly determine the occurrence and frequency of GlcNAc and GlcN units at and near the new reducing and non-reducing ends of the paCOS generated—if any [16,17]. And second, the charges and hydrophobic patches present at the ligand binding site of the receptors will determine which paCOS with defined PA will bind most efficiently and, thus, trigger a reaction. Importantly, thus, PA will crucially determine biological activities of chitosan polymers independently of whether the target hypothesis or rather the receptor hypothesis (or both) will eventually be verified.

**Figure 1:**
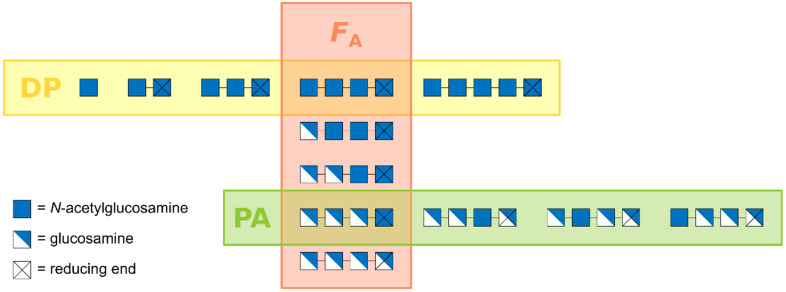
Partially acetylated chito-oligosaccharides (paCOS) are characterised by three structural parameters, namely (i) the degree of polymerisation (DP), i.e., the number of monomeric units, (ii) the fraction of acetylation (*F*_A_), i.e., the relative abundance of GlcNAc units, and (iii) the pattern of acetylation (PA), i.e., the sequence of GlcNAc and GlcN units.

Clearly then, the above mentioned 2D matrix will need to be expanded to a 3D matrix, including the influence of PA on physico-chemical properties and biological activities [1,5,6]. This 3D matrix will mark the transition from today’s second-generation defined chitosans to tomorrow’s third-generation designer chitosans. Third-generation chitosans will be defined not only in their DP and *F*_A_, but also in their PA. If we want to more fully understand molecular structure-function relationships and cellular modes of action for each of the many biological activities reported or suggested in literature, we will need fully defined paCOS, i.e., paCOS with known DP, *F*_A_, and PA (Figure 1) [18]. Such paCOS can best be produced using regio-selective chitin deacetylases, as described in this review.

## 2. Regio-Selective Chitin de-*N*-Acteylases

**Figure 2:**
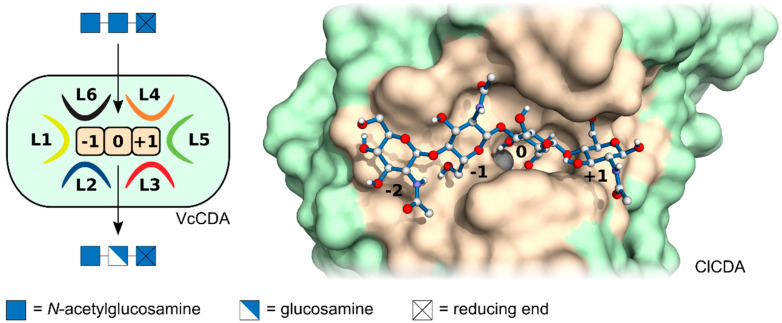
Fungal chitin deacetylases (CDA) (right), which are better known than e.g., bacterial (left) or insect CDAs, typically possess four subsite binding sites (ranging from {–2} to {+1}) within a substrate binding cleft, highlighted in beige, each of which binds one monosaccharide subunit of the substrate, a chitin or chitosan oligomer or polymer. Each subsite can have its own specificity or preference for binding a *N*-acetylglucosamine or a glucosamine unit. The substrate binding cleft, shown in beige, may be delineated by six protein loops (L1 to L6) which may be flexible, allowing an induced fit of the enzyme. Left, schematic representation of the bacterial VcCDA from *Vibrio* for which the surrounding loops where first described; right, 3D-model of the fungal ClCDA from the *Colletotrichum* with bound substrate, chitin tetraose.

Chitin deacetylases (CDAs) catalyse the hydrolytic cleavage of the acetamido bond in GlcNAc units within chitin and chitosan oligomers and polymers, deacetylating them to yield GlcN units. They belong to carbohydrate esterase family CE4, and their three-dimensional structures as well as the mode of their catalytic reaction have been described in several recent reviews [19,20,21,22]. Like many other polysaccharide modifying enzymes, such as chitinases and chitosanases [16,17,23,24,25], CDAs possess a substrate binding site consisting of several subsites, each of which binds a single monosaccharide unit of the substrate [26,27,28]. The catalytic subsite, i.e., the one binding the GlcNAc unit that will be deacetylated to yield a GlcN unit, is defined as subsite {0}, the subsite(s) ‘left’ of it, i.e., towards the non-reducing end of the substrate, are numbered as {−1}, {−2}, etc., while those ‘right’ of subsite {0}, i.e., towards the reducing end of the substrates, are {+1} etc. (Figure 2) [26]. Crystal structures of several fungal CDAs suggest that their substrate binding site comprises four subsites, ranging from {−2} to {+1} [20,28,29], while some bacterial CDAs appear to have less subsites [29], and one insect CDA has been shown to possess a larger number of subsites [30].

Like in any other enzyme, the molecular fine structure of the substrate binding site of a CDA determines its substrate specificity or preference. Two different structural features appear to govern the mode of substrate binding in CDAs, and thus their regio-selectivity, namely the position and flexibility of loops surrounding the binding site, and the specificity or preference for GlcNAc versus GlcN binding at the different subsites. The subsite capping model, which was developed based on a series of crystal structures of the *Vibrio cholerae* CDA in different states of substrate binding, describes a set of six loops surrounding the active site and suggests an induced fit model for substrate binding [29]. These loops determine the preferred positioning of the substrate within the binding site. This brings the different subunits of the substrate into close proximity to the amino acids forming the substrate binding cleft, including the catalytically active amino acid residues at subsite {0}. A partial or full negative charge in the substrate binding cleft may favour, a positive one disfavour the binding of a GlcN unit at that particular position, while the binding of a GlcNAc unit can be expected to require more space and to be supported by the presence of a hydrophobic pocket to accommodate the terminal methyl group of the acetamido side chain. In fact, the first chitosan (rather than chitin) deacetylase has recently been described, which favours GlcN over GlcNAc at subsite {−1}, and thus partially acetylated chitosan over chitin as a substrate [27].

Recent advances in the heterologous expression of genes coding for CDAs [31] and in the quantitative mass spectrometric sequencing of paCOS [32] are currently leading to an increasing number of CDAs with known subsite binding preferences and, hence, known products when acting on fully acetylated chitin oligomers (An) as substrates (Table 1). These enzymes can be used for the production of the fully defined paCOS required for advancing our understanding of molecular structure-function relationships and cellular modes of action of biologically active, partially acetylated chitosans. The portfolio of defined paCOS that can be produced using CDAs has been drastically increased with the realisation that these enzymes can also be used to catalyse the reverse reaction, i.e., the *N*-acetylation of fully de-acetylated glucosamine oligomers (Dn) in the presence of excess amounts of acetate, while mainly retaining their regio-selectivity [33,34].

Most of the times, however, the paCOS resulting from CDA action on chitin or glucosamine oligomers will not be entirely pure so that a final chromatographic purification step is typically needed. In the following paragraphs, we will first describe the production of the substrates, i.e., fully acetylated chitin and fully deacetylated glucosamine oligomers of defined DP, followed by the enzymatic strategies that can yield fully defined dimeric to hexameric paCOS. The chapter will end with short descriptions of the state-of-the-art of paCOS analysis and purification.

## 3. Preparation of the Substrates (An and Dn)

The enzymatic production of defined paCOS using CDAs depends on the availability of suitably pure substrates, namely fully acetylated chitin oligomers and fully deacetylated glucosamine oligomers of defined DP. Different methods to this end have been described, and such oligomers are commercially available, though at rather high prices only, due to the rather expensive production and purification steps involved.

**Figure 3:**
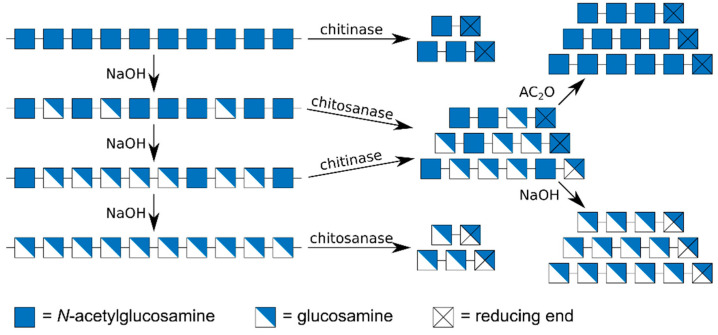
Chemo-enzymatic production of fully acetylated chitin oligomers and fully de-acetylated glucosamine oligomers. Fully acetylated chitin dimers A2 and trimers A3 can be generated from polymeric chitin by the action of a chitinase, fully de-acetylated dimers D2 and trimers D3 from polyglucosamine, itself prepared from chitin by sequential steps of alkaline de-acetylation, by the action of a chitosanase. Larger oligomers can be produced by the action of chitinase or chitosanase on partially acetylated chitosan polymers followed by alkaline de-acetylation yielding GlcN oligomers, or by chemical *N*-acetylation using acetic anhydride, yielding GlcNAc oligomers.

One approach is the partial acid or enzymatic hydrolysis of chitin to yield chitin oligomers (An), or of polyglucosamine to yield glucosamine oligomers (Dn) (Figure 3). Chitin is commercially available, and it can be converted to polyglucosamine using sequential steps of alkaline treatment [40,41]. Clearly, the purity of the polymeric substrates will determine the product quality that can be achieved. Acid hydrolysis of chitin needs to be performed carefully, e.g., using concentrated rather than dilute acids, to prevent concomitant loss of acetyl groups [42,43,44]. Enzymatic depolymerisation of chitin and polyglucosamine can be achieved using chitinases and chitosanases, respectively. Care must be taken to avoid the use of processive enzymes which would yield mostly dimeric products [23,45]. The main drawback of this approach is that both chemical and enzymatic hydrolyses are difficult to control, both proceed quickly to the production of very small, mostly monomeric (acid hydrolysis) or di- and trimeric (enzymatic hydrolysis) products [46].

An alternative approach giving more easy access to larger oligomers is the partial acid or enzymatic hydrolysis of partially acetylated chitosan polymers followed by full chemical *N*-acetylation to yield chitin oligomers (An) or by full chemical de-acetylation to yield glucosamine oligomers (Dn). The choice of polymeric substrate used, particularly its *F*_A_, and of the depolymerising agent used, particularly its sequence specificity, will determine the size range of products obtained. The glycosidic linkage of a GlcNAc residue within a chitosan polymer is more acid-labile than that of a GlcN unit so that the polymer chain will preferentially break at a GlcNAc residue during acid hydrolysis [42,44]. Consequently, the size of the oligomeric products obtained, which *cum grano salis* represent contiguous D-blocks within the original polymer chain, will increase with decreasing *F*_A_ of the chitosan used. In fact, as the distribution of GlcNAc units within commercial chitosans follows a random (Bernoulli) distribution [47,48], the size range of oligomeric products obtained can roughly be predicted as a function of the *F*_A_ of the polymer used. However, as chitosan polymer samples invariably are mixtures of molecules with different *F*_A_ so that the *F*_A_ given or measured is an average value only [49], with unknown dispersity *Đ*_FA_ [6], such a calculation can give a rough estimate only of the true DP range experimentally obtained.

The hydrolytic process can be controlled more precisely when using enzymes instead of acid. Chitinases require at least one GlcNAc unit within the chitosan polymer chain to bind and hydrolyse their substrate [50,51,52,53], while chitosanases require at least one GlcN unit [17,25]. Hence, the size of chitinase products increases, while that of chitosanase products decreases with decreasing *F*_A_ of the substrate. While the DP range of the products is more difficult to predict compared to acid hydrolysis, unless the subsite specificity of the enzyme used is precisely known, enzymatic hydrolysis can more easily yield a broader range of products, as enzymes with different levels of subsite specificities are available [17,32,54,55,56]. Clearly, the more specific the enzyme, the larger its products. In any case, the enzymatic products will be mixtures of partially acetylated oligomers (paCOS) which can be converted to fully acetylated chitin oligomers (An) by *N*-acetylation or to fully de-acetylated glucosamine oligomers (Dn) by de-acetylation, as above.

**Figure 4:**
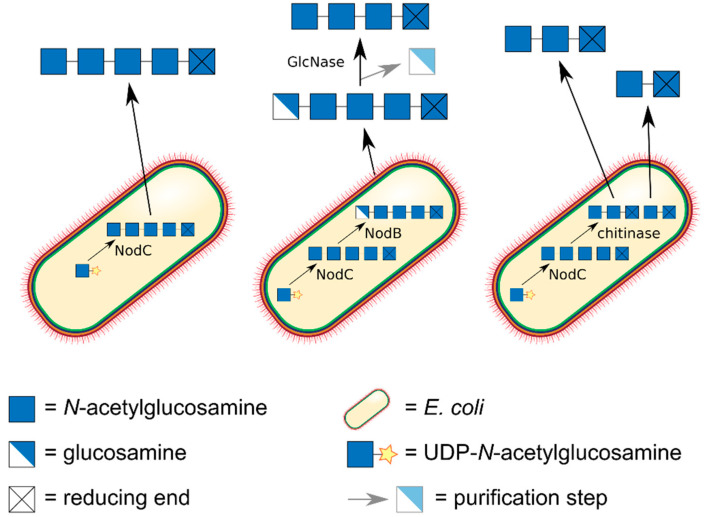
Fully acetylated chitin dimers A2, trimers A3, tetramers A4, and pentamers A5 can be produced biotechnologically in *E. coli* cell factories on kg scale. An *E. coli* strain expressing the chitin oligomer synthase NodC from *Rhizobium* yields A5. A strain expressing NodC together with NodB yields the α-mono-deacetylated pentamer DAAAA, which can be converted into A4 by GlcNase treatment to remove the deacetylated unit from the non-reducing end. A strain co-expressing NodC and a chitinase produces A3 and A2 which can be separated using chromatography.

Certainly, the most elegant, but also the most demanding approach towards production of chitin oligomers (An) is their biotechnological production in dedicated cell factories (Figure 4) [57]. As early as 1994, Geremia et al. expressed a bacterial chitin oligomer synthase, NodC from *Azorhizobium caulinodans*, in *E. coli*, driving the synthesis of chitin pentamer (A5) [58]. The beauty of this approach is its scalability: gram amounts of rather pure chitin oligomers can thus easily be produced [59]. Co-expressing a chitinase yielded chitin dimer (A2) and trimer (A3) [60], which of course could also be generated from the biotechnologically produced pentamer using the recombinant chitinase in vitro. Co-expressing the regio-selective chitin deacetylase NodB from *A. caulinodans,* which specifically deacetylates the GlcNAc unit at the non-reducing end of the chitin oligomer, yielded the α-mono-deacetylated (‘α’ indicating the non-reducing end unit) chitosan pentamer (A4D1) with the sequence DAAAA [59]. Using a GlcN-specific glucosaminidase (GlcNase) [61], this product might be converted to yield the chitin tetramer (A4) [62].

Independent of its route of synthesis, the resulting chitin or glucosamine oligomers will need to be purified, to remove oligomers of smaller or larger size than the targeted DP as well as contaminants left over from the synthesis. This is particularly crucial if the oligomers are destined to be used later in bioactivity studies. Purification can best be achieved using chromatographic steps, such as size exclusion chromatography, as detailed at the end of this chapter. Another important issue is the stability of the oligomers which easily oxidize, visible as a yellowing of the sample. This can be prevented by storage under anaerobic conditions or in the presence of salt (which, however, may present difficulties in later enzymatic conversion and, particularly, in bioassays) [63]. An added problem for storage arises for fully or partially de-acetylated oligomers, especially those of low DP, as the free amino groups may react with the aldehyde group of the reducing ends of the oligomers, in a Maillard-type reaction which equally leads to browning of the samples [64]. This can be inhibited by strongly diluting the sample before freeze drying it.

## 4. paCOS Nomenclature

In this chapter, we will adopt a widely used, but not yet formalized nomenclature for the description of paCOS. First, as already introduced above, we will abbreviate GlcNAc units as ‘A’ and GlcN units as ‘D’. Second, we will use the form ‘AnDm’ to indicate the number of A and D units within the oligomer, without making any statement concerning the sequence of the monomeric units. Thus, ‘AnDm’ includes all paCOS of DP = n + m and *F*_A_ = n/(n + m). A4D1, e.g., would indicate a paCOS (or a paCOS mixture) of DP 5 and *F*_A_ 0.8, with no information given on the PA or sequence of the units. Third, individual positions within the oligomer will be denoted with Greek letters, ‘α‘ denoting the non-reducing end unit and ‘ω’ the reducing end unit of the paCOS. Consequently, the unit next-to the non-reducing end unit is named ‘β’, followed by ‘γ’, while the unit next to the reducing end unit is named ‘ψ’, preceded by ‘χ’. Finally, to indicate the full sequence of a paCOS, the units are listed from the non-reducing to the reducing end, e.g., ‘ADAAA’ for a β-mono-deacetylated pentameric paCOS. As a consequence, ‘AD’ indicates the α-acetylated (or ω-deacetylated) dimer GlcNAc-GlcN, while ‘A1D1’ summarily denotes AD and the α-deacetylated (or ω-acetylated) dimer GlcN-GlcNAc, i.e., it can either indicate one of them (without indicating which one) or a mixture of both of them.

## 5. Preparation of paCOS Dimers

**Figure 5:**
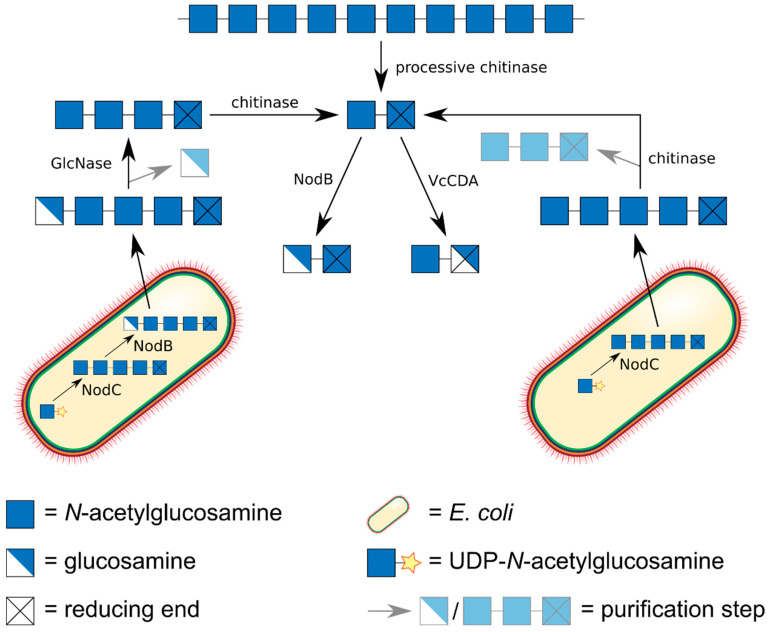
Biotechnological preparation of the two dimeric paCOS DA and AD. DA can be prepared from chitobiose AA by the action of the CDA NodB from *Rhizobium*. AD can be prepared from chitobiose AA by the action of VcCDA from *Vibrio*. The substrate chitobiose AA can be prepared from polymeric chitin or from the chitin pentaose A5 or from the chitin tetraose A4 by the action of a (preferentially processive) chitinase such as SmChiB; the oligomeric substrates being produced by *E. coli* cell factories expressing the chitin oligomer synthase NodC from *Rhizobium* to yield A5 or NodC together with NodB to yield the α-mono-deacetylated pentamer DAAAA followed by GlcNase treatment to remove the deacetylated unit from the non-reducing end. In each case, the chitobiose has to be purified using liquid chromatography.

There are only two possible dimeric paCOS, namely AD and DA, both of which can easily be produced from chitobiose (A2) using regio-selective, bacterial CDAs. Also, production of the substrate chitobiose is comparatively simple, as it is the main product of chitinase hydrolysis of chitin, particularly if a processive chitinase is used [45,65]. Alternatively, biotechnologically produced chitin pentamer (A5) or preferably, chitin tetramer (A4) may be used as a substrate. Unless the tetramer is used, this approach will yield a mixture of chitin dimer and trimer so that chromatographic purification is required. Chitobiose can be converted to the dimer DA by the use of NodB from any rhizobial strain [35], while the CDA from *Vibrio cholerae* or *Vibrio parahaemolyticus*, VcCDA or VpCDA, will yield the dimer AD [36,66] (Figure 5).

## 6. Preparation of paCOS Trimers

**Figure 6:**
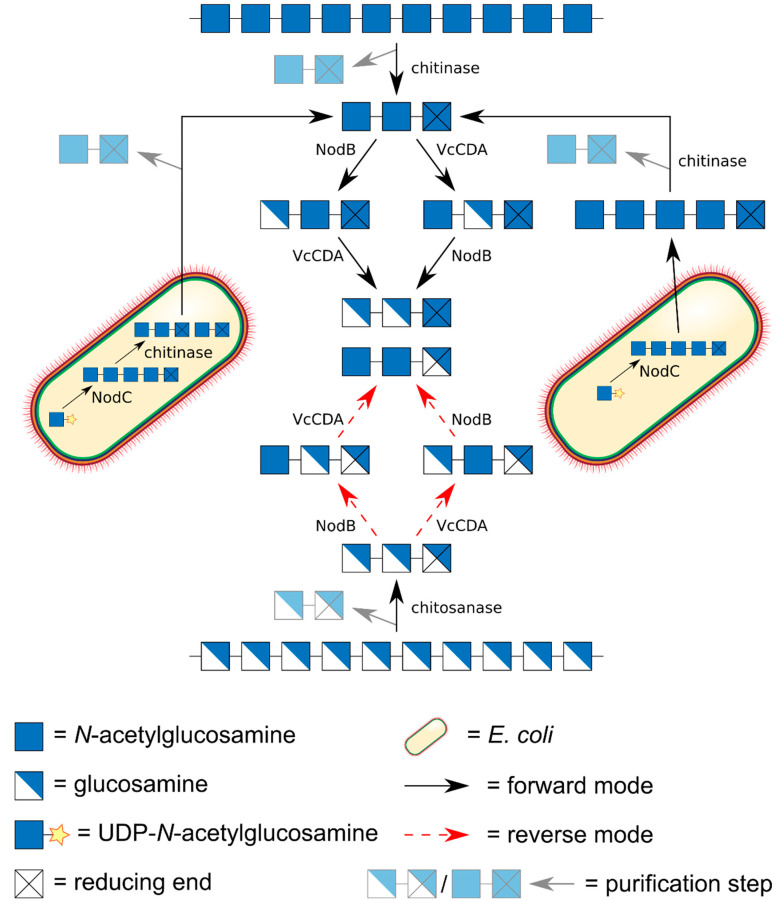
Biotechnological preparation of the six trimeric paCOS DAA, ADA, AAD, DDA, DAD, and ADD. DAA and ADA can be prepared from chitotriose AAA by the action of the CDA NodB from *Rhizobium* and VcCDA from *Vibrio*, respectively. DDA can then be produced by subsequent treatment with the other enzyme. Similarly, ADD and DAD can be prepared from DDD by the action of these two enzymes acting in reverse mode in the presence of excess amounts of acetate, and AAD can then be produced by subsequent reverse action of the other enzyme. The substrates for these reactions, AAA and DDD, can be prepared from polymeric chitin or polyglucosamine by the action of a chitinase or chitosanase, respectively. Alternatively, chitotriose can be produced by an *E. coli* cell factory strain expressing NodC from *Rhizobium*, yielding chitin pentaose which can then be cleaved, in vivo or in vitro, by a chitinase, yielding chitobiose and chitotriose. In each case, the trimeric product has to be purified using liquid chromatography.

While there are only two possible dimeric paCOS, there are already 2^3^ − 2 = 6 different trimeric paCOS which can all be produced using the two bacterial CDAs described above, NodB and VcCDA, which act on the non-reducing α-end unit or the β-unit next to the non-reducing end of their substrates, respectively (Figure 6). The fully acetylated substrate A3 can be produced as described above for chitobiose production, namely by chitinase digestion of chitin or biotechnologically produced A5, followed by SEC purification. Similarly, the fully deacetylated glucosamine trimer (D3) can best be produced by chitosanase digestion of polyglucosamine, yielding mostly GlcN dimer (D2) and trimer (D3) which can be separated using SEC [67,68].

There are three different mono-deacetyated trimeric paCOS (A2D1). The trimer DAA can easily be produced from the fully acetylated substrate A3 by the α-action of NodB, while the β-acting VcCDA will yield the trimer ADA when acting on the same substrate [69]. The third, ω-mono-de-acetylated paCOS, AAD, is more difficult to produce as no CDA with regio-selectivity for the GlcNAc unit at the reducing end has been described so far. However, if NodB and VcCDA are used in reverse on the fully deacetylated glucosamine trimer D3 in the presence of excess amounts of acetate, they retain their regio-selectivity, so that their combined use will yield AAD [34].

Similarly, there are three different mono-acetylated paCOS (A1D2) which can be produced in mirror routes to the ones described above. Reverse α-action of NodB on D3 will yield ADD, while reverse β-action of VcCDA will yield DAD [34]. The third, ω-mono-acetylated trimer, DDA, can be produced by the joint forward activity of the two enzymes on the fully acetylated trimer A3 [34].

## 7. Preparation of paCOS Tetramers

**Figure 7:**
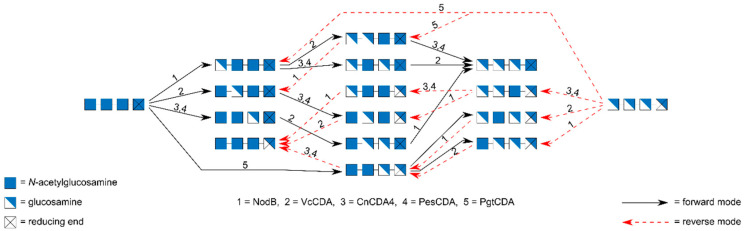
Biotechnological preparation of the fourteen possible tetrameric paCOS using CDAs in forward or reverse mode on the fully acetylated or fully de-acetylated tetramers AAAA and DDDD, respectively, as described previously [34]. Both substrates can be prepared biotechnologically in good yields, AAAA by an *E. coli* cell factory expressing NodC and NodB to produce DAAAA followed by GlcNase treatment to yield AAAA, DDDD by digestion of polyglucosamine using an engineered chitosanase unable to cleave tetrameric substrates [70]. Both AAAA and DDDD need to be purified before converting them to paCOS using CDAs. Only five different CDAs are required, acting alone or in sequence, to produce all tetramers. For some of the tetramers, alternative routes exist and typically, chromatographic purification is required for the production of pattern-pure paCOS.

There are already 2^4^ − 2 = 14 different possible tetrameric paCOS, and all of them can be produced using a combination of different CDAs acting in forward or reverse mode [34] (Figure 7). Briefly, different CDAs are known which are highly specific in their first deacetylation step so that three of the four mono-deacetylated tetramers are easily produced, only the one with a GlcN unit at its reducing end, AAAD, is more demanding to produce, and the same is true for the production of the mono-acetylated tetramers, using the same enzymes in reverse. All of the six double-acetylated/double-deacetylated tetramers can be produced in not more than two enzymatic steps. In most cases, chromatographic purification is required to obtain pattern-pure tetramers.

The tetrameric substrates required, A4 and D4, are more difficult to produce than the smaller substrates required for the production of the dimeric and trimeric paCOS. Both the fully acetylated chitin tetramer A4 and the fully de-acetylated glucosamine tetramer D4 can be produced from tetrameric paCOS generated by the action of chitinase or chitosanase on partially acetylated chitosan polymers, followed by SEC purification. Alternatively, A4 can be produced by GlcNase digestion of the α-mono-deacetylated pentameric paCOS DAAAA, synthesized itself by an *E. coli* cell factory expressing NodC and NodB from *Rhizobium*, as described above. There is also an alternative route to the fully de-acetylated tetramer D4 using a mutant of the chitosanase *Bsp*CsnMN which has been engineered using site directed mutagenesis, to reduce its ability to hydrolyse the tetramer which consequently is obtained as a product of polyglucosamine digestion, of course also requiring SEC purification [70].

## 8. Preparation of paCOS Pentamers and Hexamers

**Figure 8:**
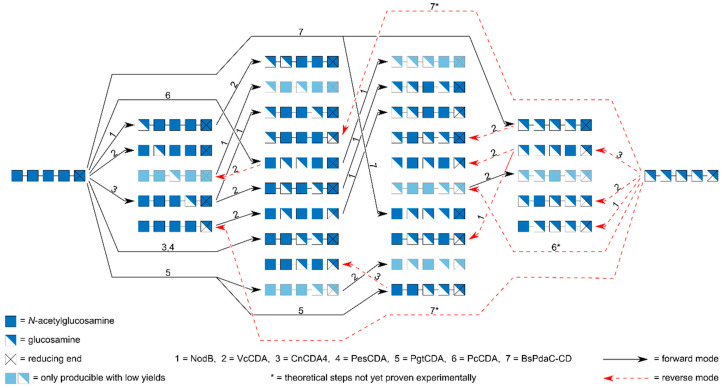
Experimentally proven and theoretical production routes for all thirty possible pentameric paCOS using known CDAs, alone or in combination, in forward or reverse mode. This overview shows the most feasible production route for each pentamer, but alternative routes exist for many of them. Since it has not yet been shown that PcCDA and BsPdaC also work in reverse mode, these hypothetical steps are marked with an asterisk.

There are 2^5^ − 2 = 30 different pentameric paCOS and 2^6^ − 2 = 62 different hexameric paCOS. Not all of them can yet be produced using enzymatic routes. Also, their chromatographic purification is more demanding and still partly impossible. However, a surprisingly large number of paCOS pentamers and hexamers can be produced using the rather few CDAs currently available whose regio-selectivity is known in sufficient detail, as shown in Figure 8 for the pentamers. While it is theoretically possible to produce all pentamers in this way using seven different CDAs, some will be difficult to produce and their yields can be expected to be low, and most of them will require chromatographic purification. Also, for two of these enzymes, PcCDA and BsPdaC [38,39], *N*-acetylating activity in the presence of excess amounts of acetate has not yet been shown experimentally, but is assumed hypothetically here. For many of the pentamers, different production routes can be designed but for clarity, only the most feasible one is indicated in the figure. For some products, alternative routes may give higher yields than the route shown here, but we opted for experimentally proven routes over theoretically assumed ones even if the yield of the former is known to be low while that of the latter is expected to be higher. As an example, it is known that AAADD can be produced from A5 by PgtCDA as a by-product in rather low yields (main product is AADDA) [31]. The alternative and possibly higher-yielding production route for this pentamer would start with BsPdaC acting in reverse on D5 and yielding AAAAD, followed by CnCDA4-catalysed de-acetylation to yield AAADD, but the reverse action of BsPDAC has not yet been verified experimentally.

Overall, the production of the larger oligomers such as the pentamer is more demanding than that of the tetramers, and additional characteristics of the different CDAs beyond their regio-selectivity need to be taken into account. One example is the fact that some CDAs, e.g., VcCDA, prefer small substrates over larger ones. In the case of VcCDA, the mutein VcCDA K275E/H127 has been designed for higher activity on larger oligomers [71], and its use is therefore recommended in the production of pentameric (and larger) paCOS. Also, while NodB and VcCDA will only de-acetylate or *N*-acetylate a single unit even on larger oligomers, CnCDA4 and PesCDA can deacetylate further positions, thus decreasing the yield for their single step products. This also decreases the purity of the product, since the intermediate products are often not pattern-pure and only if they differ in their reducing end, they can be separated chromatographically even when having the same *F*_A_ (see below). For the same reason, also ADDDA and DAAAD will not be pattern-pure, since they are not the final product of BsPdaC, and they cannot (yet) be purified chromatographically from their by-products.

In some cases, the sequence in which the enzymes are used also makes a difference when these larger paCOS with DP > 4 are targeted. As an example, we mentioned above that three of the four mono-deacetylated tetramers can be produced rather easily, since NodB, VcCDA, and CnCDA4 or PesCDA prefer to bind either the reducing or the non-reducing end of their substrate at a specific position within their substrate binding groove, so that specific subsites that are important for substrate binding are occupied. On larger oligomers, however, other effects also play a role. The chitosan deacetylase CnCDA4, for example, starts de-acetylating the fully acetylated pentamer A5 at the position next to the reducing end of the substrate, yielding AAADA. However, as the enzyme highly prefers a de-acetylated GlcN unit at subsite {−1}, it is impossible to predict whether it would produce ADADA or ADDAA when acting on ADAAA as a substrate [27]. Thus, when targeting the paCOS ADADA, it is preferable to first use CnCDA4 on A5 yielding AAADA followed by VcCDA to yield the targeted ADADA, rather than vice versa.

While enzymatic production of all pentameric paCOS is already feasible using the limited number of CDA available which are well enough characterised concerning their precise mode of action, it would highly profit from a few additional CDAs possessing as yet non-described specificities. As an example, the middle position of the pentamer is difficult to de-acetylated or *N*-acetylate since none of the CDAs described so far are reported to specifically act on this (γ- or χ-) position. The middle position can only be targeted indirectly using first PcCDA (to yield ADDAA from A5, or DAADD from D5), then VcCDA (converting ADDAA to AADAA in reverse action, or DAADD to DDADD in forward action). If we had a CDA which specifically acted on the middle positions, some of the pentameric paCOS would be easier to produce with better yields. A similar problem occurs for the reducing end, as none of the CDAs described today acts exclusively on that ω-position. Therefore, AAAAD needs to be produced in reverse mode by BsPdaC, a step not yet proven experimentally. As mentioned above, this step would also be a welcome first step in the production of AAADD (by CnCDA4 acting on AAAAD) and ADAAD (by VcCDA acting on AAAAD) in possibly good yields.

Of course, as for the shorter paCOS, the fully acetylated and fully de-acetylated substrates A5 and D5 need to be produced first. This can be achieved as described above for the tetramers, i.e., by enzymatic digestion of partially acetylated chitosan polymers followed by chemical *N*-acetylation or de-acetylation. The chitin pentamer A5 can also be produced biotechnologically in the *E. coli* cell factory expressing NodC [59], and this A5 might also be fully de-acetylated using alkali to yield D5.

As expected, production of defined, pattern-pure paCOS with a DP > 5 is even more demanding. In principle, hexameric paCOS can be produced in the same manner of combining the different CDAs described for the pentameric paCOS production. In this way, more than half of the 62 possible hexameric paCOS can theoretically be produced, although some of them probably only at very low yields. And of course, chromatographic separation will become even more difficult or, rather, impossible.

Here, some of the difficulties already described for the production of pentameric paCOS become even more problematic. Again, it is difficult to attack the two middle positions of the hexamer. Let us look at the production of AADAAA. As described by Hembach et al. (2020) [27], ADDAAA can be produced from A6 by the sequential action of the mutein VcCDA K275E/H127 yielding ADAAAA followed by CnCDA4 (or possibly directly by PcCDA acting on A6). Then, ADDAAA needs to be *N*-acetylated again using the VcCDA mutein to produce the targeted AADAAA. De-acetylation of the other middle position to obtain AAADAA might be possible using PgtCDA as this is the dominating pattern within the mono-deacetylated products of this enzyme. However, PgtCDA very quickly deacetylates a second position, yielding only very low amounts of mono-deacetylated products in general. In addition, as described for the pentameric paCOS, the mixture of mono-deacetylated hexamers cannot be fully separated by HPLC, so that a mixture of A5D1 paCOS will be obtained, with AAADAA being the most prominent, but not the only one.

While with the pentamers, the reducing end can be modified using either PgtCDA or BsPdaC in normal or reverse mode, this becomes more challenging with the hexamers, where PgtCDA was not reported to produce AAAADD, but rather AAADDA or AAADDD. It might still be possible to use BsPdaC in reverse mode, though this step has not yet been proven experimentally even on short oligomers, and it can be expected to become more challenging with larger oligomers, since effects such as product inhibition or problems caused by the high salt concentration required for *N*-acetylation may lead to strongly reduced enzyme activity towards the end of the reaction when the fifth acetate needs to be attached to yield the targeted AAAAAD.

## 9. Mass Spectrometric Analysis of paCOS

**Figure 9:**
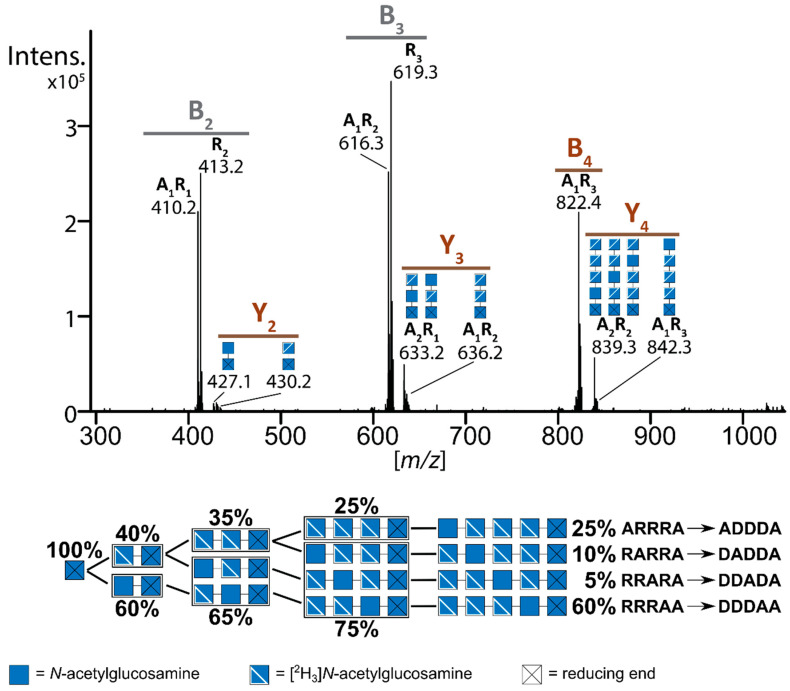
Quantitative MS/MS sequencing of a mixture of isotopically labelled chitosan pentamers (A2D3). A partially acetylated chitosan polymer was enzymatically digested, the free amino groups of GlcN (D) residues within the oligomeric products obtained were acetylated using [^2^H_6_]-acetic anhydride, resulting in [^2^H_3_]*N*-acetylglucosamine (R), and their reducing ends were ^18^O-labelled. The labelled oligomers were separated using UHPLC hydrophilic interaction liquid chromatography (HILIC) and analyzed using ESI-MS/MS. The sequences (PA) of the chitosan oligomers in the triple-de-acetylated pentamers A2D3 was determined by using the relative abundances, shown in the boxes below the spectrum, of the fragment ions (denoted as B- and Y-ions according to nomenclature created by Domon and Costello in 1988 [74]), highlighted in brown.

The structural analysis of paCOS has recently been revolutionized by the use of mass spectrometry (MS) which has a number of advantages over the previously required NMR spectroscopy [32,72,73]. NMR can only solve the sequence of purified paCOS and, concerning full sequencing, is limited to oligomers of low DP ≤ 4, as it informs mainly on the α- and ω-end units and their immediately neighbouring β- and ψ-units. In contrast, mass spectrometry can precisely determine the molecular weight of paCOS even in complex mixtures, directly informing about both DP and *F*_A_, and MS^2^ techniques can then be used to determine PA also, thus giving full structural information. Recently, a sophisticated quantitative sequencing method involving isotopic labelling of oligomers using deuterated acetic anhydride for *N*-acetylation of paCOS has been described (Figure 9) [32]. 

The thus obtained fully re-acetylated chitin oligomers, in which the deuterated acetyl groups mark the positions of originally de-acetylated GlcN units in the paCOS, can then be fragmented and quantitatively analysed, avoiding problems arising from differences in e.g., fragmentation or ionization of GlcN and GlcNAc units. In this way, the paCOS present in mixtures, e.g., obtained as a result of enzymatic hydrolysis of chitosan polymers or enzymatic de-acetylation or *N*-acetylation of GlcNAc and GlcN oligomers, respectively, can be identified and quantified, as long as the mixtures are not too complex. The rather low aqueous solubility of chitin oligomers limits this analysis to paCOS with DP ≤ 6. An added advantage of MS over NMR is that the latter requires multiple mg-amounts of sample, while the former can be performed on sub-µg samples.

## 10. Chromatographic Purification of paCOS

**Figure 10:**
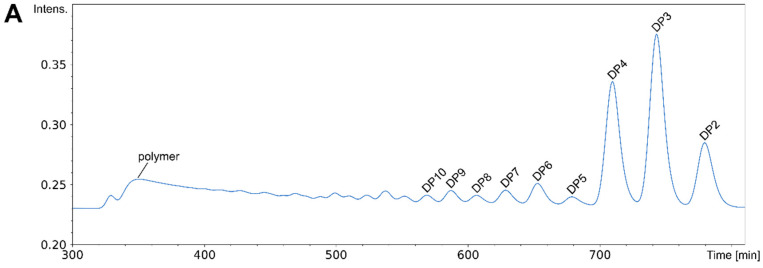

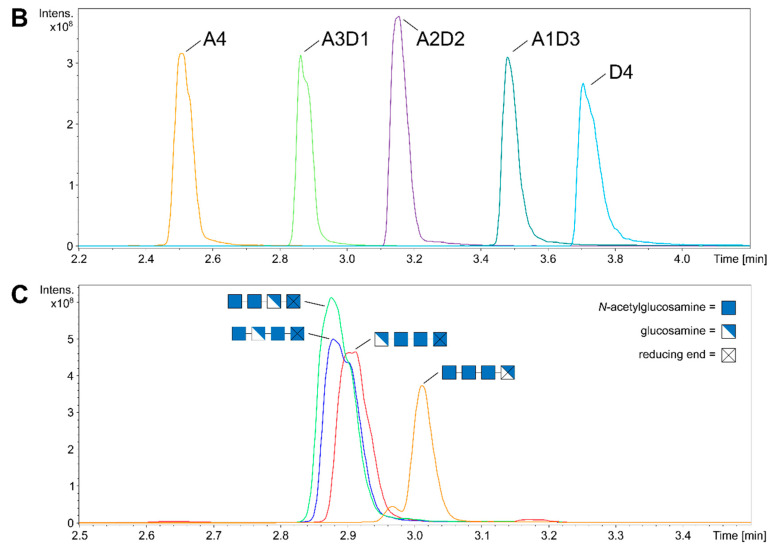
Chromatographic separation of different paCOS. (**A**) SEC-chromatogram of the separation of a mixture of chitosans ranging from dimers to polymer according to their degree of polymerisation (DP). (**B**) Overlay-HILIC chromatogram of HILIC-purified tetrameric paCOS with different fractions of acetylation (*F*_A_). (**C**) Overlay-HILIC-chromatogram of HILIC-purified mono-deacetylated tetrameric paCOS with different patterns of acetylation (PA).

Typically, both the fully acetylated and the fully de-acetylated substrates An and Dn as well as the paCOS produced from them using CDAs in forward or reverse mode need to be purified using chromatography to yield single paCOS [34,69]. The most straightforward separation is that according to DP which can be achieved using size exclusion chromatography (SEC) [23,69]. If sufficiently long columns are used, baseline separation of oligomers up to DP ca. 15 can be achieved. The molecular weights of the four different dimers are sufficiently different (DD = 340 Da, AD = DA = 382 Da, AA = 424 Da) so that they can be partially separated; the heaviest dimer (AA) is sufficiently lighter than the lightest trimer (DDD = 501 Da) so that the dimer and trimer peaks do not overlap; the six different trimers (D3 = 501 Da, A1D2 = 543 Da, A2D1 = 585 Da, A3 = 627 Da) are not sufficiently different to be separated from each other. As a consequence, chitin and chitosan oligomers (with the exception of dimers) are separated solely according to their DP on currently used SEC columns (Figure 10A).

A disadvantage of SEC is that typically, rather high salt concentrations are needed in the elution buffer to prevent unwanted complex formation of the oligomers and unwanted interactions of the oligomers with the chromatography matrix. The use of volatile salts such as ammonium acetate allows to remove them prior to e.g., bioassays, but several sequential freeze-drying steps are required for complete salt removal. To prevent browning of the samples, the use of high volumes of water is required in each step, making the process slow.

Fully acetylated chitin oligomers have also been separated by using reverse phase chromatography (RPC) [75] or normal phase chromatography (NPC) [76,77]. In contrast to SEC, water can be used as the eluent here, so that no salt removal is required after separation. Fully de-acetylated glucosamine oligomers can also be separated using cation exchange chromatography (CEC), but again with the disadvantage that rather high salt concentrations are required in the eluent [78].

Separating paCOS of equal DP according to their *F*_A_ is more demanding but can also be achieved, using either CEC, hydrophobic interaction chromatography (HIC), or hydrophilic interaction liquid chromatography (HILIC). The latter has been shown to even separate some paCOS of equal DP and equal *F*_A_, but different PA [34,73] (Figure 10B,C).

## 11. Conclusions and Outlook

Recombinant regio-selective CDAs acting in forward or reverse mode on DP-pure chitin or glucosamine oligomers followed by chromatographic purification can yield a plethora of partially acetylated chito-oligosaccharides with fully defined architecture in terms of DP, *F*_A_, and PA. These can then be used in a broad range of cellular bio-assays to quantify e.g., their antibacterial, antifungal, or antiviral activities, their plant growth promoting, disease resistance inducing, and stress tolerance increasing activities, their anti- or pro-inflammatory, anti-oxidative or anti-tumour activities and many many more. This will allow to fine-tune our current understanding of molecular structure-function relationships of partially acetylated chitosans, but will also enable studies on their cellular and physiological mode of action. Clearly, such studies will open up a whole new dimension of chitosan research and development and will pave the way for third-generation chitosans with known and defined patterns of acetylation. A few studies already hint at the crucial role of PA on properties and bioactivities of chitosans, as recently reviewed [6,18], and a very recent publication unequivocally proved for the first time that the α-mono-acetylated chitosan tetramer with the GlcNAc unit at its non-reducing end had strong disease resistance priming activities in rice cells, while the priming activities sequentially decreased in the other three isomeric paCOS, and the ω-mono-acetylated one with the GlcNAc unit at the reducing end was completely inactive [79]. Importantly, this priming-active tetramer possessed no elicitor activity, i.e., it prepares plant cells to efficiently react to even low concentrations of a resistance reaction-inducing elicitor that would not trigger a naïve cell, but it does not induce such an energy-consuming and potentially self-destructing cellular resistance reaction by itself. Such an elicitor-inactive, but priming-active substance is the ideal lead compound for the development of a plant “vaccination” strategy—an urgent necessity in our globalised world in which diseases travel freely and increasingly threaten a safe supply of healthy food and sustainable biomaterials and biologics.

Today, not all paCOS can be produced by the enzymatic approach using CDAs. Our improving abilities to heterologously produce these enzymes can be expected to yield more CDAs with differing regio-selectivities, which we can also characterise more precisely given the recent rapid development of analytical tools. This will enable us to make use of nature’s biodiversity in our attempts to provide full sets of fully defined paCOS. At the same time, our increasing knowledge of the structures of CDA enzymes, their subsite specificities or preferences, and their enzymatic modes of action will allow us to design and engineer CDA enzymes with new or more precise regio-selectivities to increase and complement the natural biodiversity [27]. In parallel to these developments, strategies for the chemical synthesis of defined paCOS are also improving, and solid-state synthesis is promising to yield paCOS of every structure at will, even for higher DP, possibly up to DP 10 or even higher [80]. Once these will become available, they will be a more than welcome complementation to the enzymatically produced paCOS. Studies of structure-function relationships have an inherent problem caused by the potentially extremely high bioactivity of signal molecules, necessitating extremely pure test compounds to avoid false conclusions based on bioactivities of minor contaminants. This problem occurs independent of the route of synthesis of the signal compound studied, be it chemical or enzymatic. However, given that possible contaminants can be expected to differ between the two routes, comparison of bioactivities of e.g., paCOS produced by chemical synthesis versus enzymatic modification is the safest way to come to conclusive results, as previously done for other bioactive oligosaccharides [81,82,83,84]. The two methods also complement each other in yet another way. While chemical synthesis might eventually turn out to be more flexible in yielding every wanted paCOS, particularly solid-state synthesis cannot easily be scaled to yield larger amounts of products. In contrast, enzymatic synthesis is scalable, both in vitro and in vivo, and cell factories may eventually yield kg amounts of defined paCOS, potentially making the process economically feasible [57]. And finally, we can envisage the development of chemo-enzymatic approaches in which e.g., chemically synthesised paCOS are elongated using glycosyl transferases [85] or joint to yield larger paCOS using glycosyl hydrolases [86] or engineered glyco-synthases [87,88]. The world of third-generation chitosans is only beginning to emerge, but already promises a new era of chitosan research and development of chitosan-based products.

## Figures and Tables

**Table 1:** Products of regio-selective CDAs which have been characterized in detail.

Enzyme	Step	Substrate					**Reference**
		AA	AAA	AAAA	AAAAA	AAAAAA	
NodB	1	DA	DAA	DAAA	DAAAA	DAAAAA	[35]
VcCDA	1	AD	ADA	ADAA	ADAAA	ADAAAA	[36]
CnCDA4	1	-	(ADA)	AADA	AAADA	AAAADA	[27]
	2		-	AADD	AADDA	AAADDA	
	3			-	-	AADDDA	
PesCDA	1	-	-	AADA	AA[AD]A	AA[A2D1]A	[37]
	2			-	AADDA	AA[A1D2]A	
	3				-	AADDDA	
PgtCDA	1	p.n.d.	p.n.d.	AADA	AAADA (AADAA)	AAADAA (AADAAA)	[31]
	2			AADD	AADDA (AAADD)	AAADDA (AADDAA)	
	3			-	AADDD	AADDDA (AAADDD)	
	4				-	AADDDD	
PcCDA	1	a.n.d.	-	A3D1	ADAAA	a.n.d.	[38]
	2			A2D2	ADDAA		
BsPdaC-CD	1	-	p.n.d.	ADAA AADA	AAADA (AADAA)	a.n.d.	[39]
	2			ADDA (DADA)	AADDA (ADADA)		
	3			DDDA	ADDDA		
	4			-	DDDDA		

() = by-product; p.n.d. = pattern not determined; a.n.d. = activity not determined; [] = pattern not completely resolved; - = no activity; A = GlcNAc; D = GlcN.

## Abbreviations

DP	Degree of Polymerisation
*F*_A_	Fraction of Acetylation
PA	Pattern of Acetylation
paCOS	partially acetylated Chitosan Oligosaccharides
CDA	Chitin Deacetylase

## References

[1] El Gueddari N.E., Moerschbacher B.M. (2004). A bioactivity matrix for chitosans as elicitors of disease resistance reactions in wheat. Adv. Chitin Sci..

[2] El Gueddari N.E., Kolkenbrock S., Schaaf A., Chilukoti N., Brunel F., Gorzelanny C., Fehser S., Chachra S., Bernard F., Nampally M. (2014). Chitin and chitosan modifying enzymes: Versatile novel tools for the analysis of structure-function relationship of partially acetylaed chitosans. Adv. Chitin Sci..

[3] Kauss H., Jeblick W., Domard A. (1989). The degrees of polymerization and N-acetylation of chitosan determine its ability to elicit callose formation in suspension cells and protoplasts of *Catharanthus roseus*. Planta.

[4] Vander P., Vårum K.M., Domard A., El Gueddari N.E., Moerschbacher B.M. (1998). Comparison of the Ability of Partially N-Acetylated Chitosans and Chitooligosaccharides to Elicit Resistance Reactions in Wheat Leaves. Plant Physiol..

[5] Moerschbacher B.M., Gnanamanickam S.S., Balasubramanian R., Anand N. (2005). Bio-activity matrices of chitosans in plant protection. Emerging Trends in Plant-Microbe Interactions.

[6] Wattjes J., Sreekumar S., Richter C., Cord-Landwehr S., Singh R., El Gueddari N.E., Moerschbacher B.M. (2020). Patterns matter part 1: Chitosan polymers with non-random patterns of acetylation. React. Funct. Polym..

[7] Thadathil N., Suresh P. (2014). Recent developments in chitosanase research and its biotechnological applications: A review. Food Chem..

[8] Arnold N.D., Brück W.M., Garbe D., Brück T.B. (2020). Enzymatic Modification of Native Chitin and Conversion to Specialty Chemical Products. Mar. Drugs.

[9] Júnior E.N., El Gueddari N.E., Moerschbacher B.M., Franco T.T. (2012). Growth rate inhibition of phytopathogenic fungi by characterized chitosans. Braz. J. Microbiol..

[10] Lacombe-Harvey M.È., Fukamizo T., Gagnon J., Ghinet M.G., Dennhart N., Letzel T., Brzezinski R. (2009). Accessory active site residues of *Streptomyces* sp. N174 chitosanase. FEBS J..

[11] Ghinet M.G., Roy S., Poulin-Laprade D., Lacombe-Harvey M.È., Morosoli R., Brzezinski R. (2010). Chitosanase from Streptomyces coelicolorA3(2): Biochemical properties and role in protection against antibacterial effect of chitosan. Biochem. Cell Biol..

[12] Miya A., Albert P., Shinya T., Desaki Y., Ichimura K., Shirasu K., Narusaka Y., Kawakami N., Kaku H., Shibuya N. (2007). CERK1, a LysM receptor kinase, is essential for chitin elicitor signaling in Arabidopsis. Proc. Natl. Acad. Sci. USA.

[13] Kaku H., Nishizawa Y., Ishii-Minami N., Akimoto-Tomiyama C., Dohmae N., Takio K., Minami E., Shibuya N. (2006). Plant cells recognize chitin fragments for defense signaling through a plasma membrane receptor. Proc. Natl. Acad. Sci. USA.

[14] Gubaeva E., Gubaev A., Melcher R.L.J., Cord-Landwehr S., Singh R., El Gueddari N.E., Moerschbacher B.M. (2018). Slipped Sandwich Model for Chitin and Chitosan Perception in Arabidopsis. Mol. Plant-Microbe Interact..

[15] Fuchs K., Gloria Y.C., Wolz O., Herster F., Sharma L., Dillen C.A., Täumer C., Dickhöfer S., Bittner Z., Dang T. (2018). The fungal ligand chitin directly binds TLR 2 and triggers inflammation dependent on oligomer size. EMBO Rep..

[16] Sørbotten A., Horn S.J., Eijsink V.G.H., Vårum K.M. (2004). Degradation of chitosans with chitinase B from *Serratia marcescens*. FEBS J..

[17] Weikert T., Niehues A., Cord-Landwehr S., Hellmann M.J., Moerschbacher B.M. (2017). Reassessment of chitosanase substrate specificities and classification. Nat. Commun..

[18] Cord-Landwehr S., Richter C., Wattjes J., Sreekumar S., Singh R., Basa S., El Gueddari N.E., Moerschbacher B.M. (2020). Patterns matter part 2: Chitosan oligomers with defined patterns of acetylation. React. Funct. Polym..

[19] Aragunde H., Biarnés X., Planas A. (2018). Substrate Recognition and Specificity of Chitin Deacetylases and Related Family 4 Carbohydrate Esterases. Int. J. Mol. Sci..

[20] Grifoll-Romero L., Pascual S., Aragunde H., Biarnés X., Planas A. (2018). Chitin Deacetylases: Structures, Specificities, and Biotech Applications. Polymers.

[21] Zhao Y., Park R.-D., Muzzarelli R.A.A. (2010). Chitin Deacetylases: Properties and Applications. Mar. Drugs.

[22] Tsigos I., Martinou A., Kafetzopoulos D., Bouriotis V. (2000). Chitin deacetylases: New, versatile tools in biotechnology. Trends Biotechnol..

[23] Horn S.J., Sorbotten A., Synstad B., Sikorski P., Sorlie M., Varum K.M., Eijsink V.G.H. (2006). Endo/exo mechanism and processivity of family 18 chitinases produced by *Serratia marcescens*. FEBS J..

[24] Fukamizo T., Ohkawa T., Ikeda Y., Goto S. (1994). Specificity of chitosanase from *Bacillus pumilus*. Biochim. Biophys. Acta (BBA) Protein Struct. Mol. Enzym..

[25] Hirano K., Watanabe M., Seki K., Ando A., Saito A., Mitsutomi M. (2012). Classification of Chitosanases by Hydrolytic Specificity toward N1, N4-Diacetylchitohexaose. Biosci. Biotechnol. Biochem..

[26] Tokuyasu K., Mitsutomi M., Yamaguchi I., Hayashi K., Mori Y. (2000). Recognition of Chitooligosaccharides and TheirN-Acetyl Groups by Putative Subsites of Chitin Deacetylase from a Deuteromycete, *Colletotrichum lindemuthianum*. Biochemistry.

[27] Hembach L., Bonin M., Gorzelanny C., Moerschbacher B.M. (2020). Unique subsite specificity and potential natural function of a chitosan deacetylase from the human pathogen *Cryptococcus neoformans*. Proc. Natl. Acad. Sci. USA.

[28] Blair D.E., Hekmat O., Schüttelkopf A.W., Shrestha B., Tokuyasu K., Withers S.G., Van Aalten D.M.F. (2006). Structure and Mechanism of Chitin Deacetylase from the Fungal Pathogen *Colletotrichum lindemuthianum*. Biochemistry.

[29] Andrés E., Albesa-Jové D., Biarnés X., Moerschbacher B.M., Guerin M.E., Planas A. (2014). Structural Basis of Chitin Oligosaccharide Deacetylation. Angew. Chem. Int. Ed..

[30] Liu L., Zhou Y., Qu M., Qiu Y., Guo X., Zhang Y., Liu T., Yang J., Yang Q. (2019). Structural and biochemical insights into the catalytic mechanisms of two insect chitin deacetylases of the carbohydrate esterase 4 family. J. Biol. Chem..

[31] Naqvi S., Cord-Landwehr S., Singh R., Bernard F., Kolkenbrock S., El Gueddari N.E., Moerschbacher B.M. (2016). A Recombinant Fungal Chitin Deacetylase Produces Fully Defined Chitosan Oligomers with Novel Patterns of Acetylation. Appl. Environ. Microbiol..

[32] Cord-Landwehr S., Ihmor P., Niehues A., Luftmann H., Moerschbacher B.M., Mormann M. (2017). Quantitative Mass-Spectrometric Sequencing of Chitosan Oligomers Revealing Cleavage Sites of Chitosan Hydrolases. Anal. Chem..

[33] Tokuyasu K., Ono H., Hayashi K., Mori Y. (1999). Reverse hydrolysis reaction of chitin deacetylase and enzymatic synthesis of β-d-GlcNAc-(1→4)-GlcN from chitobiose. Carbohydr. Res..

[34] Hembach L., Cord-Landwehr S., Moerschbacher B.M. (2017). Enzymatic production of all fourteen partially acetylated chitosan tetramers using different chitin deacetylases acting in forward or reverse mode. Sci. Rep..

[35] John M., Rohrig H., Schmidt J., Wieneke U., Schell J. (1993). Rhizobium NodB protein involved in nodulation signal synthesis is a chitooligosaccharide deacetylase. Proc. Natl. Acad. Sci. USA.

[36] Li X., Wang L.-X., Wang X., Roseman S. (2007). The Chitin Catabolic Cascade in the Marine Bacterium *Vibrio Cholerae*: Characterization of a Unique Chitin Oligosaccharide Deacetylase. Glycobiology.

[37] Cord-Landwehr S., Melcher R.L.J., Kolkenbrock S., Moerschbacher S., Moerschbacher B.M. (2016). A chitin deacetylase from the endophytic fungus *Pestalotiopsis* sp. efficiently inactivates the elicitor activity of chitin oligomers in rice cells. Sci. Rep..

[38] Aranda-Martinez A., Grifoll-Romero L., Aragunde H., Sancho-Vaello E., Biarnés X., Lopez-Llorca L.V., Planas A. (2018). Expression and specificity of a chitin deacetylase from the nematophagous fungus *Pochonia chlamydosporia* potentially involved in pathogenicity. Sci. Rep..

[39] Grifoll-Romero L., Sainz-Polo M.A., Albesa-Jové D., Guerin M.E., Biarnés X., Planas A. (2019). Structure-function relationships underlying the dual N-acetylmuramic and N-acetylglucosamine specificities of the bacterial peptidoglycan deacetylase PdaC. J. Biol. Chem..

[40] No H.K., Meyers S.P., Muzzarelli R.A.A., Peter M.G. (1997). Preperation of chitin and chitosan. Chitin Handbook.

[41] Roberts G.A.F. (1998). Chitosan production routes and their role in determining the structure and properties of the product. Adv. Chitin Sci..

[42] Varum K. (2001). Acid hydrolysis of chitosans. Carbohydr. Polym..

[43] Einbu A., Vårum K.M. (2007). Depolymerization and De-N-acetylation of Chitin Oligomers in Hydrochloric Acid. Biomacromolecules.

[44] Einbu A., Grasdalen H., Vårum K.M. (2007). Kinetics of hydrolysis of chitin/chitosan oligomers in concentrated hydrochloric acid. Carbohydr. Res..

[45] Vaaje-Kolstad G., Horn S.J., Sørlie M., Eijsink V.G.H. (2013). The chitinolytic machinery of Serratia marcescens—A model system for enzymatic degradation of recalcitrant polysaccharides. FEBS J..

[46] Kidibule P.E., Santos-Moriano P., Jiménez-Ortega E., Ramírez-Escudero M., Limón M.C., Remacha M., Plou F.J., Sanz-Aparicio J., Fernández-Lobato M. (2018). Use of chitin and chitosan to produce new chitooligosaccharides by chitinase Chit42: Enzymatic activity and structural basis of protein specificity. Microb. Cell Factories.

[47] Kumirska J., Weinhold M.X., Steudte S., Thöming J., Brzozowski K., Stepnowski P. (2009). Determination of the pattern of acetylation of chitosan samples: Comparison of evaluation methods and some validation parameters. Int. J. Biol. Macromol..

[48] Vårum K.M., Anthonsen M.W., Grasdalen H., Smidsrød O. (1991). 13C-N.m.r. studies of the acetylation sequences in partially N-deacetylated chitins (chitosans). Carbohydr. Res..

[49] Cord-Landwehr S., Niehues A., Wattjes J., Moerschbacher B.M., van den Broek L.A.M., Boeriu C.G. (2019). New developments in the analysis of partially acetylated chitosan polymers and oligomers. Chitin and Chitosan.

[50] Van Aalten D.M.F., Komander D., Synstad B., Gaseidnes S., Peter M.G., Eijsink V.G.H. (2001). Structural insights into the catalytic mechanism of a family 18 exo-chitinase. Proc. Natl. Acad. Sci. USA.

[51] Ohno T., Armand S., Hata T., Nikaidou N., Henrissat B., Mitsutomi M., Watanabe T. (1996). A modular family 19 chitinase found in the prokaryotic organism *Streptomyces griseus* HUT 6037. J. Bacteriol..

[52] Fukamizo T., Koga D., Goto S. (1995). Comparative Biochemistry of Chitinases—Anomeric Form of the Reaction Products. Biosci. Biotechnol. Biochem..

[53] Sasaki C., Vårum K.M., Itoh Y., Tamoi M., Fukamizo T. (2006). Rice chitinases: Sugar recognition specificities of the individual subsites. Glycobiology.

[54] Regel E.K., Weikert T., Niehues A., Moerschbacher B.M., Singh R. (2018). Protein-engineering of chitosanase from *Bacillus* sp. MN to alter its substrate specificity. Biotechnol. Bioeng..

[55] Bußwinkel F., Goñi O., Cord-Landwehr S., O’Connell S., Moerschbacher B.M. (2018). Endochitinase 1 (Tv-ECH1) from Trichoderma virens has high subsite specificities for acetylated units when acting on chitosans. Int. J. Biol. Macromol..

[56] Aam B.B., Heggset E.B., Norberg A.L., Sørlie M., Vårum K.M., Eijsink V.G.H. (2010). Production of Chitooligosaccharides and Their Potential Applications in Medicine. Mar. Drugs.

[57] Naqvi S., Moerschbacher B.M. (2015). The cell factory approach toward biotechnological production of high-value chitosan oligomers and their derivatives: An update. Crit. Rev. Biotechnol..

[58] Geremia R.A., Mergaert P., Geelen D., Van Montagu M., Holsters M. (1994). The NodC protein of *Azorhizobium caulinodans* is an N-acetylglucosaminyltransferase. Proc. Natl. Acad. Sci. USA.

[59] Samain E., Drouillard S., Heyraud A., Driguez H., Geremia R.A. (1997). Gram-scale synthesis of recombinant chitooligosaccharides in *Escherichia coli*. Carbohydr. Res..

[60] Cottaz S., Samain E. (2005). Genetic engineering of *Escherichia coli* for the production of NI,NII-diacetylchitobiose (chitinbiose) and its utilization as a primer for the synthesis of complex carbohydrates. Metab. Eng..

[61] Tanaka T., Fukui T., Atomi H., Imanaka T. (2003). Characterization of an Exo-β-d-Glucosaminidase Involved in a Novel Chitinolytic Pathway from the Hyperthermophilic Archaeon *Thermococcus kodakaraensis* KOD1. J. Bacteriol..

[62] Hamer S.N., Moerschbacher B.M., Kolkenbrock S. (2014). (Stephan) Enzymatic sequencing of partially acetylated chitosan oligomers. Carbohydr. Res..

[63] Ajandouz E.H., Tchiakpe L., Ore F.D., Benajiba A., Puigserver A. (2001). Effects of pH on Caramelization and Maillard Reaction Kinetics in Fructose-Lysine Model Systems. J. Food Sci..

[64] Chung Y.C., Tsai C.-F., Li C.F. (2006). Preparation and characterization of water-soluble chitosan produced by Maillard reaction. Fish. Sci..

[65] Waghmare S.R., Ghosh J.S. (2010). Chitobiose production by using a novel thermostable chitinase from *Bacillus licheniformis* strain JS isolated from a mushroom bed. Carbohydr. Res..

[66] Kadokura K., Sakamoto Y., Saito K., Ikegami T., Hirano T., Hakamata W., Oku T., Nishio T. (2007). Production of a recombinant chitin oligosaccharide deacetylase from *Vibrio parahaemolyticus* in the culture medium of *Escherichia coli* cells. Biotechnol. Lett..

[67] Kurakake M., Yo-U S., Nakagawa K., Sugihara M., Komaki T. (2000). Properties of chitosanase from *Bacillus cereus* S1. Curr. Microbiol..

[68] Jung W., Park R.-D. (2014). Bioproduction of Chitooligosaccharides: Present and Perspectives. Mar. Drugs.

[69] Hamer S.N., Cord-Landwehr S., Biarnés X., Planas A., Waegeman H., Moerschbacher B., Kolkenbrock S. (2015). Enzymatic production of defined chitosan oligomers with a specific pattern of acetylation using a combination of chitin oligosaccharide deacetylases. Sci. Rep..

[70] Gercke D., Regel E., Singh R., Moerschbacher B.M. (2019). Rational protein design of *Bacillus* sp. MN chitosanase for altered substrate binding and production of specific chitosan oligomers. J. Biol. Eng..

[71] Pascual S., Planas A. (2018). Screening Assay for Directed Evolution of Chitin Deacetylases: Application to *Vibrio cholerae* Deacetylase Mutant Libraries for Engineered Specificity. Anal. Chem..

[72] Haebel S., Bahrke A.S., Peter† M.G. (2007). Quantitative Sequencing of Complex Mixtures of Heterochitooligosaccharides by vMALDI-Linear Ion Trap Mass Spectrometry. Anal. Chem..

[73] Tang M.-C., Nisole A., Dupont C., Pelletier J.N., Waldron K.C. (2011). Chemical profiling of the deacetylase activity of acetyl xylan esterase A (AxeA) variants on chitooligosaccharides using hydrophilic interaction chromatography–mass spectrometry. J. Biotechnol..

[74] Domon B., Costello C.E. (1988). A systematic nomenclature for carbohydrate fragmentations in FAB-MS/MS spectra of glycoconjugates. Glycoconj. J..

[75] Lopatin S., Ilyin M., Pustobaev V., Bezchetnikova Z., Varlamov V., Davankov V. (1995). Mass-Spectrometric Analysis of N-Acetylchitooligosaccharides Prepared through Enzymatic Hydrolysis of Chitosan. Anal. Biochem..

[76] Li X.J., Piao X.S., Kim S.W., Liu P., Wang L., Shen Y.B., Jung S.C., Lee H.S. (2007). Effects of Chito-Oligosaccharide Supplementation on Performance, Nutrient Digestibility, and Serum Composition in Broiler Chickens. Poult. Sci..

[77] Sashiwa H., Fujishima S., Yamano N., Kawasaki N., Nakayama A., Muraki E., Sukwattanasinitt M., Pichyangkura R., Aiba S.-I. (2003). Enzymatic production of N-acetyl-d-glucosamine from chitin. Degradation study of N-acetylchitooligosaccharide and the effect of mixing of crude enzymes. Carbohydr. Polym..

[78] Li K., Liu S., Xing R., Yu H., Qin Y., Li R., Li P. (2013). High-resolution separation of homogeneous chitooligomers series from 2-mers to 7-mers by ion-exchange chromatography. J. Sep. Sci..

[79] Basa S., Nampally M., Honorato T., Das S.N., Podile A.R., El Gueddari N.E., Moerschbacher B.M. (2020). The Pattern of Acetylation Defines the Priming Activity of Chitosan Tetramers. J. Am. Chem. Soc..

[80] DelBianco M., Kononov A., Poveda A., Yu Y., Diercks T., Jiménez-Barbero J., Seeberger P.H. (2018). Well-Defined Oligo- and Polysaccharides as Ideal Probes for Structural Studies. J. Am. Chem. Soc..

[81] Darvill A.G., Albersheim P. (1984). Phytoalexins and their Elicitors—A Defense Against Microbial Infection in Plants. Annu. Rev. Plant Physiol..

[82] Ossowski P., Garegg P.J., Lindberg B. (1983). Syntheses of a Branched Hepta and an Octassaccharide with Phytoalexin-Elicitor Activity. Angew. Chem. Int. Ed..

[83] Aly M.R., Ibrahim E.-S.I., El Ashry E.S.H., Schmidt R.R. (2001). Synthesis of chitotetraose and chitohexaose based on dimethylmaleoyl protection. Carbohydr. Res..

[84] Kuyama H., Nukada T., Ito Y., Nakahara Y., Ogawa T., Nakahara Y., Nakahara Y. (1993). Stereocontrolled synthesis of chitosan dodecamer. Carbohydr. Res..

[85] Slámová K., Krejzová J., Marhol P., Kalachova L., Kulik N., Pelantová H., Cvačka J., Křen V. (2015). Synthesis of Derivatized Chitooligomers using Transglycosidases Engineered from the Fungal GH20 β-N-Acetylhexosaminidase. Adv. Synth. Catal..

[86] Kobayashi S., Kiyosada A.T., Shoda S.-I. (1996). Synthesis of Artificial Chitin: Irreversible Catalytic Behavior of a Glycosyl Hydrolase through a Transition State Analogue Substrate. J. Am. Chem. Soc..

[87] Honda Y., Fushinobu S., Hidaka M., Wakagi T., Tsuruta T., Taniguchi H., Kitaoka M. (2008). Alternative strategy for converting an inverting glycoside hydrolase into a glycosynthase. Glycobiology.

[88] Alsina C., Faijes M., Planas A. (2019). Glycosynthase-type GH18 mutant chitinases at the assisting catalytic residue for polymerization of chitooligosaccharides. Carbohydr. Res..

